# Protective effect of Tao Hong Si Wu Decoction against inflammatory injury caused by intestinal flora disorders in an ischemic stroke mouse model

**DOI:** 10.1186/s12906-024-04417-1

**Published:** 2024-03-18

**Authors:** Lijuan Zhang, Sujun Xue, Changyi Fei, Chao Yu, Jingjing Li, Yumeng Li, Ni Wang, Furui Chu, Lingyu Pan, Xianchun Duan, Daiyin Peng

**Affiliations:** 1 Department of Pharmacy, The First Affiliated Hospital of Anhui University of Chinese Medicine, Hefei, 230031 China; 2 School of Pharmacy, Anhui University of Chinese Medicine, Hefei, 230012 China; 3 Key Laboratory of Chinese Medicinal Formula Research, Anhui University of Chinese Medicine, Hefei, 230012 China

**Keywords:** THSWD, Ischemic stroke, Intestinal flora, Inflammation, 16SrDNA

## Abstract

**Background and aims:**

Recent studies have shown that intestinal flora are involved in the pathological process of ischemic stroke (IS). The potential protective effect of the traditional Chinese prescription, Tao Hong Si Wu Decoction (THSWD), against inflammatory injury after IS and its underlying mechanisms of action were investigated in the current study.

**Methods:**

Fifty SPF(Specefic pathogen Free) male C57 mice were randomly assigned to sham operation, model, THSWD low-dose (6.5 g/kg), medium-dose (13 g/kg) and high-dose (26 g/kg) groups (10 mice per group). Mouse models of transient middle cerebral artery occlusion were prepared via thread embolism. Neurological function score, hematoxylin-eosin (HE) staining, immunohistochemistry, enzyme-linked immunosorbent assay (ELISA), 16S ribosomal DNA (rDNA) sequencing, quantitative reverse transcription PCR (qRT-PCR) and other methods were employed to elucidate the underlying molecular mechanisms.

**Results:**

Notably, THSWD induced a reduction in the neurological function score (*P* < 0.01) and neuronal injury in brain tissue, increase in protein expression of Claudin-5 and zonula occludens-1 (ZO-1) in brain tissue(*P* < 0.01), and decrease in serum lipopolysaccharide (LPS)(*P* < 0.01), diamine oxidase (DAO)(*P* < 0.01) and D-lactic acid(*P* < 0.01, *P* < 0.05) levels to a significant extent. THSWD also inhibited the levels of tumor necrosis factor-α (TNF-α)(*P* < 0.01) and interleukin − 1β (IL-1β)(*P* < 0.01) in brain tissue, and increased alpha and beta diversity in ischemic stroke mice, along with a certain reversal effect on different microflora. Finally, THSWD inhibited the polarization of microglia cells(*P* < 0.01) and decreased the protein and gene expression of toll-like receptor-4 (TLR-4)(*P* < 0.01, *P* < 0.05) and nuclear factor kappa B (NF-κB)(*P* < 0.01) in brain tissue.

**Conclusion:**

Our data indicate that THSWD may interfere with inflammatory response in ischemic stroke by regulating intestinal flora and promoting intestinal barrier repair.

## Introduction

At present, the high morbidity and fatality rates due to stroke remain a significant clinical challenge. Both hemorrhagic and ischemic stroke pose a risk to human health, with ischemic stroke accounting for 85% of the total number of cases [1, 2]. The occurrence of ischemic stroke is attributed to interruption of the blood supply to the brain, resulting in cerebral ischemia and hypoxic lesions and necrosis of the cerebral parenchyma, which manifests clinically as neurological deficits and movement disorders [3, 4]. Inflammation is reported to play a vital role in ischemic stroke. Second ary brain injury occurs during local tissue reperfusion after cerebral ischemia, which is mainly caused by microvascular dysfunction mediated by the inflammatory reaction [5]. Thus, improvement or even reversal of the development process of ischemic stroke is a major focus of current research.

With the development of high-throughput sequencing technology and improved understanding of the microbiota-gut-brain axis theory, research attention has increasingly concentrated on the potential role of intestinal flora in neurological and mental diseases. The change of intestinal flora can affect the degree of ischemic brain injury [6]. An earlier study showed that intestinal flora of young mice could reduce the degree of brain damage after cerebral ischemia reperfusion in old mice while intestinal flora of old mice aggravated brain damage in young mice after middle cerebral artery occlusion (MCAO) [7]. The inflammatory response in ischemic stroke is closely related to imbalance of intestinal flora [8]. Intestinal flora may thus present a promising target for prevention and treatment of patients with cerebral ischemic injury [9]. Lipopolysaccharide (LPS) is a type of endotoxin from gram-negative bacteria. Intestinal flora are disordered and the level of the bacterial metabolite LPS increases after stroke. Recent studies have demonstrated that LPS can selectively increase the vascular permeability of the colon and ileum in rats, leading to destruction of the intestinal barrier and subsequent entry into the brain via the blood circulation, causing an inflammatory cascade reaction and aggravation of brain tissue injury [10]. The TLR4 receptor in brain tissue binds to LPS, triggering activation of a downstream NF-κB inflammatory signal transduction pathway, which is an important link to aggravate cerebral ischemia injury [11].

As resident macrophages in the innate immune system of the brain, microglia play a critical role in the neuroinflammatory response during cerebral ischemia [12]. In addition, intestinal microflora can affect activation of microglia and induce inflammation in vivo through the intestinal-brain axis, metabolites and other pathways, thus exacerbating cerebral ischemic injury [13]. Studies have shown that rifaximin regulates the inflammatory function of microglia by modulating intestinal microflora and short-chain fatty acids [14]. These findings support the theory that regulation of the enterocerebral axis and microglia could promote the recovery of neurological function to some extent.

THSWD is one of a number of traditional Chinese prescriptions for treatment of blood-associated diseases [15]. The TCM formula comprises six common Chinese medicinal herbs, specifically, flower of *Carthamus tinctorius* L. (Hong-hua), root of *Ligusticum chuanxiong* Hort. (Chuan-xiong), seed of *Prunus persica* (L.) Batsch (Tao-ren), root of *Paeonia lactiflora* Pall. (Bai-shao), root of *Angelica sinensis* (Oliv.) Diels (Dang-gui), and root of *Rehmannia glutinosa* (Gaertn.) DC (Shu-di) [16]. Previously, our group identified the five major components of THSWD (gallic acid, protocatechuic acid, hydroxysafflor yellow A, amygdalin, and paeoniflorin) with the aid of UPLC-QTOF-MS [17]. In recent years, our group has made some conclusions through in vivo and in vitro experiments that THSWD can reduce inflammatory injury and nerve injury by regulating mitochondrial DNA and nuclear DNA [18], mitochondrial autophagy and activation of NLRP3 inflammatory vesicles in ischemic stroke rats [19]. In vivo, it is also confirmed that THSWD inhibits the mitochondrial autophagy-NLRP12 inflammatory body pathway of brain cells to protect brain cells from injury [20]. Using the methods of network pharmacology and experimental verification, we also concluded that THSWD can protect ischemic stroke by multi-target and multi-pathway [21]. In addition, LiLi concluded that THSWD can protect cells from oxidative stress in rats with ischemic stroke through PI3K/Akt and Nrf2 signal pathways [22]. Xiaolong Lu also concluded that THSWD has a protective effect on ischemic stroke rats by inhibiting cell death [23]. However, the issue of whether THSWD protects against inflammatory injury after cerebral ischemia through regulation of intestinal flora remains to be established. Therefore, based on the flora-gut-brain axis theory, this study explored the protective effect and mechanism of THSWD on inflammatory injury after ischemic stroke, and provided a new way for the rehabilitation of ischemic stroke.

## Materials and methods

### Preparation of THSWD

THSWD consisting of Tao-ren (NO: 17,033,101), Hong-hua (NO: 17,041,401), Dang-gui (NO: 16,070,501), Chuan-xiong (NO: 17,061,601), Bai-shao (NO: 17,050,301), and Shu-di (NO: 17,042,501) at a ratio of 3:2:4:3:3:2 was purchased from Anqing Huashi Chinese Herbal Medicine Beverage Co. Ltd. (Anqing City, Anhui Province, China). THSWD was prepared and administered as reported previously [24]. Briefly, the medicinal materials were weighed out and mixed according to the required ratios. THSWD was boiled for 2 h in 10 times (v/w) water. The filtrate is collected and the residue is continued with 8 times water. The filtrate was concentrated to 2.6 g/mL by rotary evaporation.

### Animals

Male C57BL/6 mice (20–25 g) were purchased from the Hangzhou Ziyuan Experimental Animal Technology Co., Ltd(Hangzhou, China). (certificate number SCXK: Zhejiang 2019-0004). Animal experiments were approved by the Animal Ethics Committee of Anhui University of Chinese Medicine.

### Middle cerebral artery occlusion (MCAO) model

The MCAO model was generated as described previously [25]. Pentobarbital sodium has the advantages of long duration of anesthesia and little side effects, so we choose peritoneal injection of 1.5% pentobarbital sodium (50 mg/kg) to anesthetize mice. After anesthesia, the right carotid artery of male C57 mice was isolated. Following ligation of external carotid artery (ECA) and common carotid artery (CCA) and closure of the distal end of the internal carotid artery (ICA) with an artery clamp, all openings were made immediately at the bifurcation of ECA and ICA and a 6 − 0 monofilament nylon thread (diameter, 0.235 mm) inserted through the incision. Following insertion of the thread plug into ICA, the artery clip was released and the nylon thread inserted until a depth of about 10 ± 0.5 mm and the fixed nylon thread was ligated. In the sham operation group, only ECA and ICA were ligated. The model of ischemia-reperfusion injury was created by pulling out the thrombus about 3 mm after 2 h. Room temperature was maintained at 24–25 ℃ during the operation. 24 h after operation, the behavior and posture of all mice were determined using the Zea-Longa method. Higher scores equated to more severe behavioral disorder, indicative of critical neurological function defects. Scores 1–3 were included in the experimental group for follow-up analyses.

### Grouping and drug administration

The experimental groups were sham group, MCAO group, high-doseTHSWD group (2.6 g/mL), medium-dose THSWD group (1.3 g/mL) and low-dose THSWD group (0.65 g/mL). Set up 10 C57BL/6 mice in each group. THSWD treatment by gavage at a volume of 1 mL/100 g. Sham and MCAO groups were gavaged with equivalent amounts of saline for 7 days. After taking blood from eyeballs, the mice were euthanized with cervical dislocation, and then samples were taken for follow-up detection.

### Neurological score

At 7 days after treatment, Zea-Longa scoring was used to observe the behavior of mice in each group (Table 1).

**Table 1:** Zea-Longa neurobehavioral scale

Score	Performance
0	No obvious neurological impairment
1	The left forelimb is flexed, not fully extended
2	Circling to left
3	Crawling is accompanied by involuntary rotation
4	The mice could not crawl by itself

### Hematoxylin and eosin (HE) staining

At 1 h after the final administration, brain tissue was fixed with 4% paraformaldehyde solution for 24 h, paraffin-embedded, sliced, baked, dewaxed with xylene, subjected to gradient anhydrous ethanol rehydration treatment, washed with distilled water, and stained with HE. Images were obtained via optical microscopy.

### Immunohistochemistry

Brain tissue and colon were fixed with 4% paraformaldehyde solution, paraffin-embedded and sliced. Immunohistochemical staining was performed by dewaxing followed by hydration of tissue sections. Antigen retrieval was performed for 2 min, with subsequent incubation in a closed chamber at room temperature for 30 min. Sections were incubated with primary antibody at 4 °C overnight, followed by the corresponding secondary antibody (HRP label) for 50 min. Next, DAB color development, redyeing, dehydration and sealing steps were conducted. In each section, 5 cortical fields with capillaries were randomly selected under a microscope to examine the distribution of proteins along the capillaries and brown particles were positively expressed.

### Enzyme-linked immunosorbent assay

Blood was collected from eyeballs of mice,incubated for 30 min and centrifuge at 3000r/min at 4 ℃ for 10 min. The supernatant was obtained and serum LPS, DAO and D-lactic acid levels determined according to the instructions. Brain tissue was homogenized and centrifuged to obtain supernatant. Operate according to the manufacturer’s instructions. The levels of TNF-α and IL-1β were analyzed in keeping with the manufacturer’s instructions. OD values at 450 nm were measured using an enzyme labeling instrument.

### 16S ribosomal DNA (rDNA) sequencing analysis

Colons of mice were dissected, the contents squeezed into an aseptic centrifuge tube, and immediately placed on dry ice. The material was transferred to -80 ℃ for storage until experimental use. Genomic DNA was extracted and the purity and concentration of DNA were determined by agarose gel electrophoresis. The sample genomic DNA was amplified by polymerase chain reaction as a template. The primer sequences were as follows: 338 F ACTCCTACGGGAGGCAGCAG and 806R GGACTACHVGGGTWTCTAAT. Then the PCR products were mixed and purified. The library was generated using a TruSeq DNA PCR-Free Sample Preparation Kit library building toolkit, quantified using Qubit and Q-PCR, and finally sequenced with the NovaSeq600 system. Detection of intestinal flora in this experiment was entrusted to Shanghai Aoji Biotechnology Co., Ltd(Shanghai, China).

### Immunofluorescence staining

Brain tissue slices of mice from each group were roasted at 37 ℃ for 30 min and washed with PBS. Sections were sealed in 10% serum for 1 h and washed with PBS. Rabbit anti-iNOS (22226-1-AP, 1:100, Proteintech) and rabbit anti-Arg-1 (16001-1-AP, 1:500, Proteintech) were incubated overnight at 4 ℃. Next, sections were washed with PBS and incubated with FITC-labeled Alexa Fluor488 (1:1000) or Alexa Fluor594 (1:500) -coupled secondary antibody (1:1000) for 1 h away from light, followed by staining with DAPI dye solution. After DAPI solution was discarded, sections were washed with PBS. Excess water was wiped with filter paper and anti-fluorescence quenching agent added to seal the sample. Stained cells were visualized and images obtained via confocal microscopy.

### Quantitative real-time PCR(qRT-PCR)

According to the instructions, TRIzol reagent was used to extract the total DNA from the brain, and then the kit was used for reverse transcription. Real-time fluorescence quantitative PCR was performed on a BioRad IQ5 real-time fluorescence PCR instrument using the SYBR qPCR kit. The list of primers is presented in Table 2.

**Table 2:** Primer sequences list

Gene_name	Primer sequence	Product length
TLR4	Sense: GTGGCTTTATTTTGCCTTGT	171
	antisense: TTTTGCACCCTCCTTCTTT	
NF-κB	Sense: GGGGTATGCACCGTAACA	195
	antisense: GTCTCCTCCGCCTTCTG	

### Statistical analysis

SPSS 23.0 software was used for statistical analysis. The obtained data are expressed in the form of mean ± standard deviation. Independent sample T test and single factor analysis of variance for data processing. *P*-value determines the difference. Data were considered statistically significant at *P* < 0.05.

## Results

### Effect of THSWD on neurological function score in mice with ischemic stroke

**Fig. 1:**
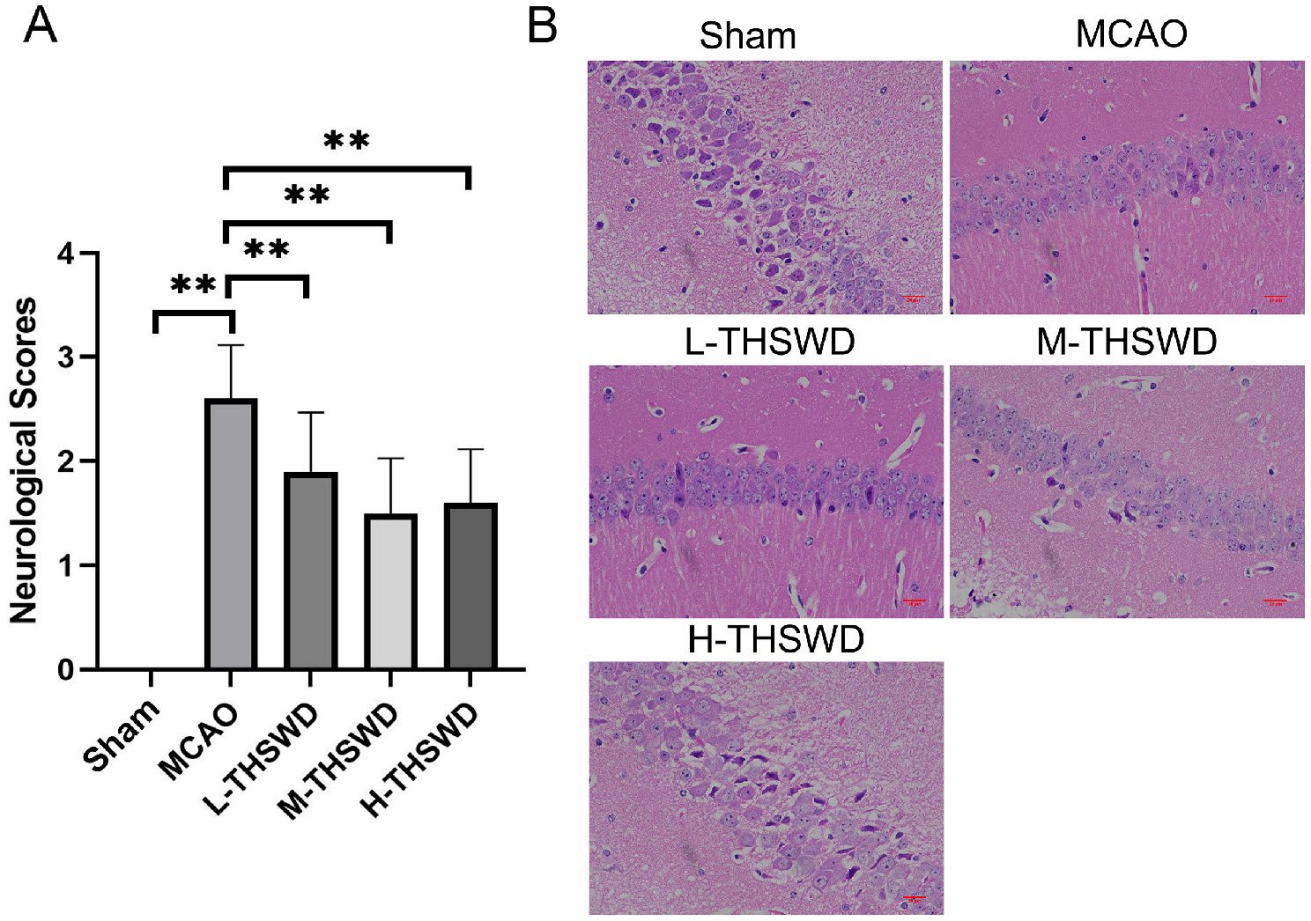
(**A**) Effect of THSWD on neurological function score in ischemic stroke mice(mean ± SD, *n* = 10). (**B**) Effect of THSWD on pathological injury of brain tissue in ischemic stroke mice (HE, Scare bar = 20 μm). ^**^P < 0.01

The neurological behaviors of mice treated with THSWD were initially evaluated. No functional loss was detected in the sham group. Compared with the sham group, the neurological function score of mice in the MCAO group was significantly increased (*P* < 0.01) while the corresponding scores of mice treated with THSWD at all three doses were significantly decreased (*P* < 0.01; Fig. 1A). The data indicate effective restoration of nerve function by THSWD.

### Effect of THSWD on pathological injury of brain tissue in mice with ischemic stroke

Hippocampal neurons in the sham group were closely arranged, abundant and normal in shape, with light cytoplasm, large and round nuclei, and no pathological changes. Compared with the sham group, the structure of neurons in the ischemic hippocampal region of mice in the MCAO group was severely damaged, along with significant loss of neuronal number, increased cytoplasmic eosinophilic staining and shrinkage. Relative to the MCAO group, the brain histomorphology of groups treated with each dose of THSWD was improved to varying degrees (Fig. 1B).

### Effect of THSWD on blood-brain barrier destruction in mice with ischemic stroke

**Fig. 2:**
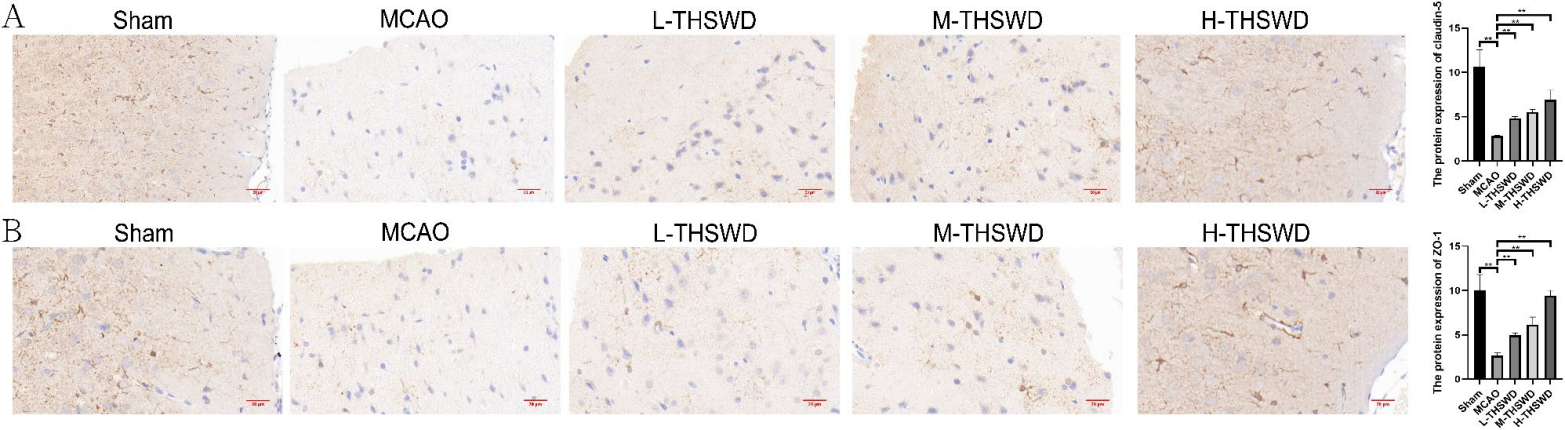
Effect of THSWD on blood-brain barrier destruction in ischemic stroke mice. (**A**) Immunohistochemical detection of claudin-5 expression in brain. (**B**) Immunohistochemical detection of ZO-1 expression in brain. Magnification: X400. Scare bar = 20 μm. ^**^P < 0.01

Compared with sham operation group, the expression of Claudin-5 and ZO-1 in brain tissue of MCAO group decreased (*P* < 0.01). Relative to the MCAO group, levels of claudin-5 and ZO-1 were increased in high- and middle-dose THSWD treatment groups (*P* < 0.01)(Fig. 2).

### Effect of THSWD on ischemic stroke-induced intestinal microbiota dysbiosis

#### Effects of THSWD on alpha and beta diversity of intestinal microflora of mice with ischemic stroke

**Fig. 3:**
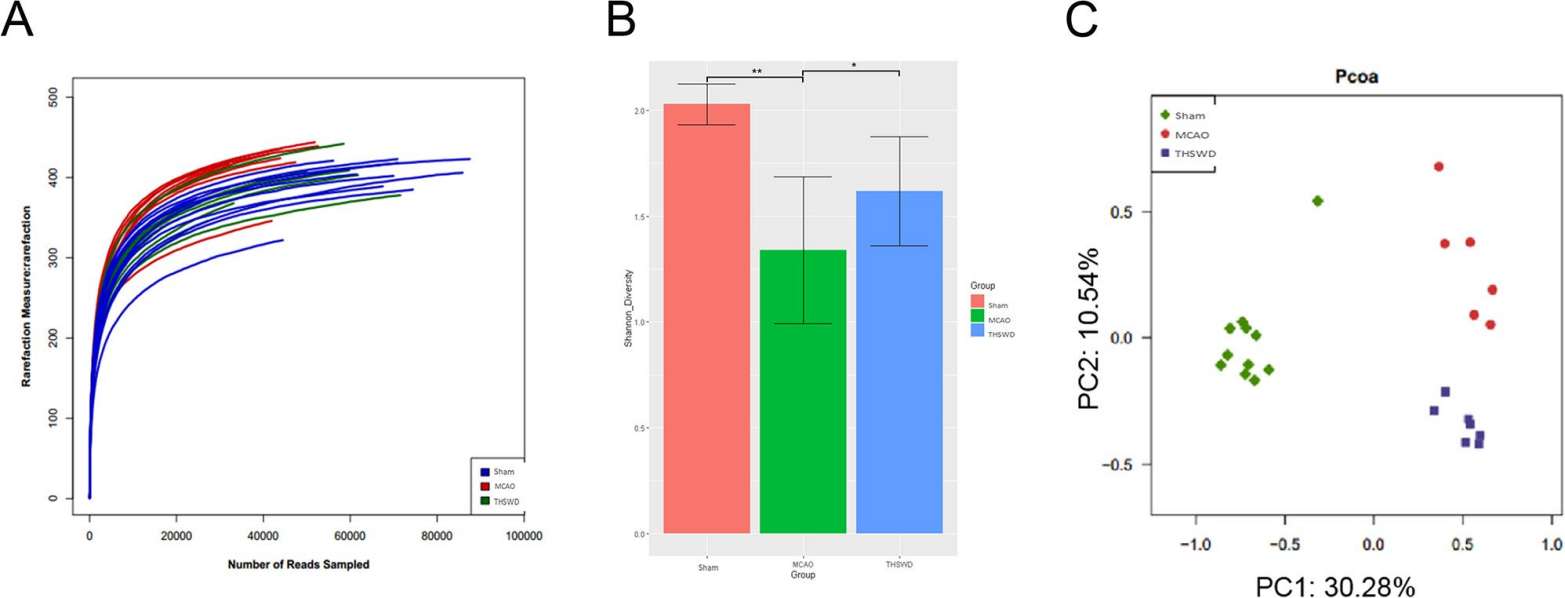
Effect of THSWD on the alpha-diversity and beta-diversity gut microbiota in ischemic stroke mice. (**A**) Dilution curve. (**B**) Shannon diversity. (**C**) Principal coordinate analysis(PCoA).

We further investigated the involvement of intestinal flora in ischemic stroke and therapeutic activity of THSWD. In pharmacodynamic experiments, high-dose treatment had the optimal repair effect on brain tissue injury. The colon contents of mice in the sham, MCAO and high-dose THSWD groups were subsequently collected for detection of changes in intestinal flora via 16S rDNA gene sequencing. The sparse curve reached a plateau, indicating that the size of the sequencing sample was sufficient (Fig. 3A). The Shannon index reflects biodiversity in the community, which is affected by both species richness and evenness. At a certain level of species richness, community diversity is greater when species evenness is higher. Relative to the sham group, the Shannon index of the MCAO group was significantly decreased (*P* < 0.01) and increased significantly following THSWD intervention (*P* < 0.05; Fig. 3B). Beta diversity measures the distance between samples according to evolutionary relationship and abundance and is applied to assess whether there are obvious differences in microflora between samples. Principal component analysis (PCA) of colonic contents revealed that the structures of intestinal flora in each group were clearly distinguishable, indicating that the flora are representative of individual environments (Fig. 3C).

#### Effect of THSWD on intestinal flora structures in mice with ischemic stroke

**Fig. 4:**
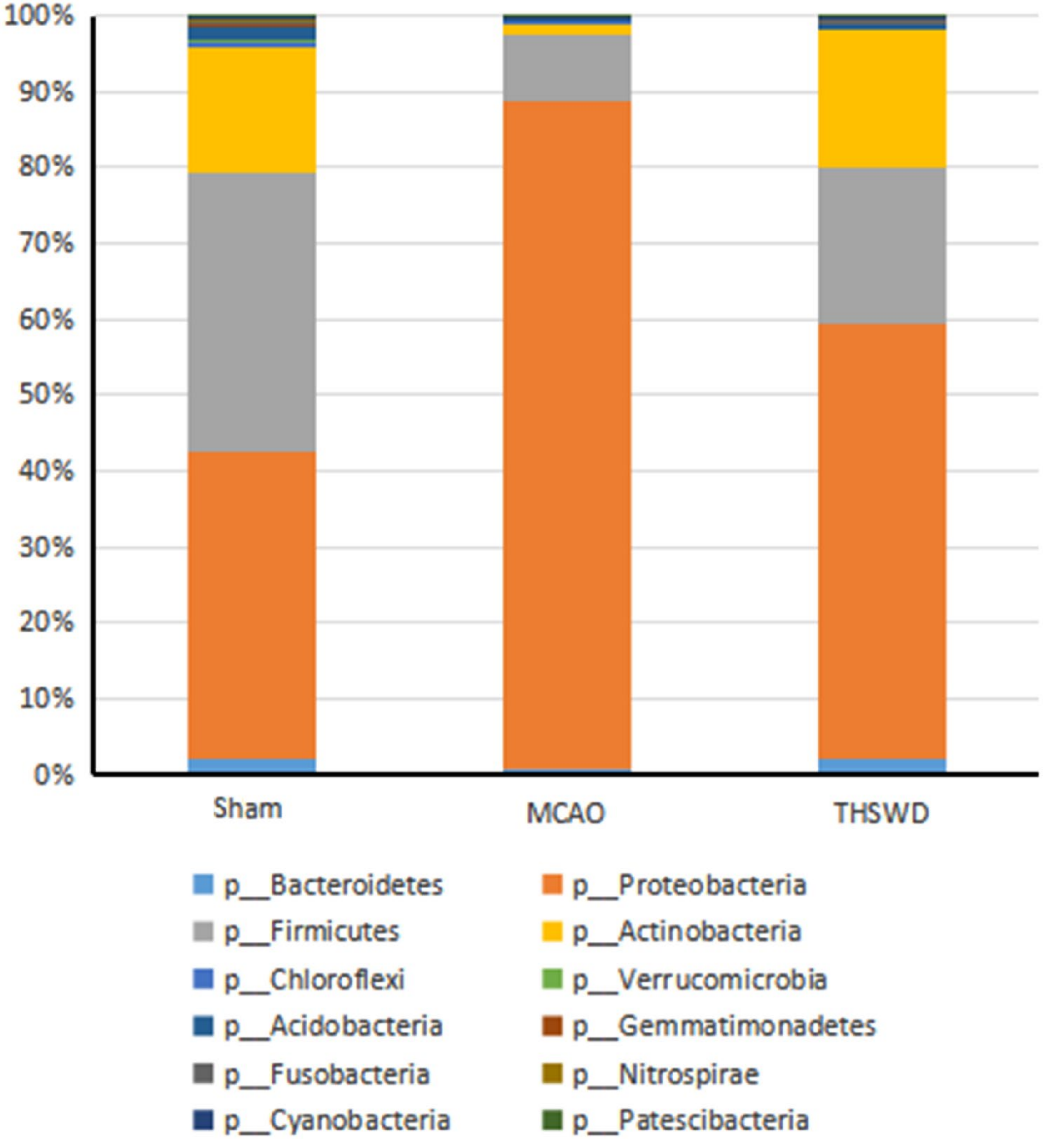
Effect of THSWD on relative abundance of gut microbiota at the level of phylum in ischemic stroke mice

**Fig. 5:**
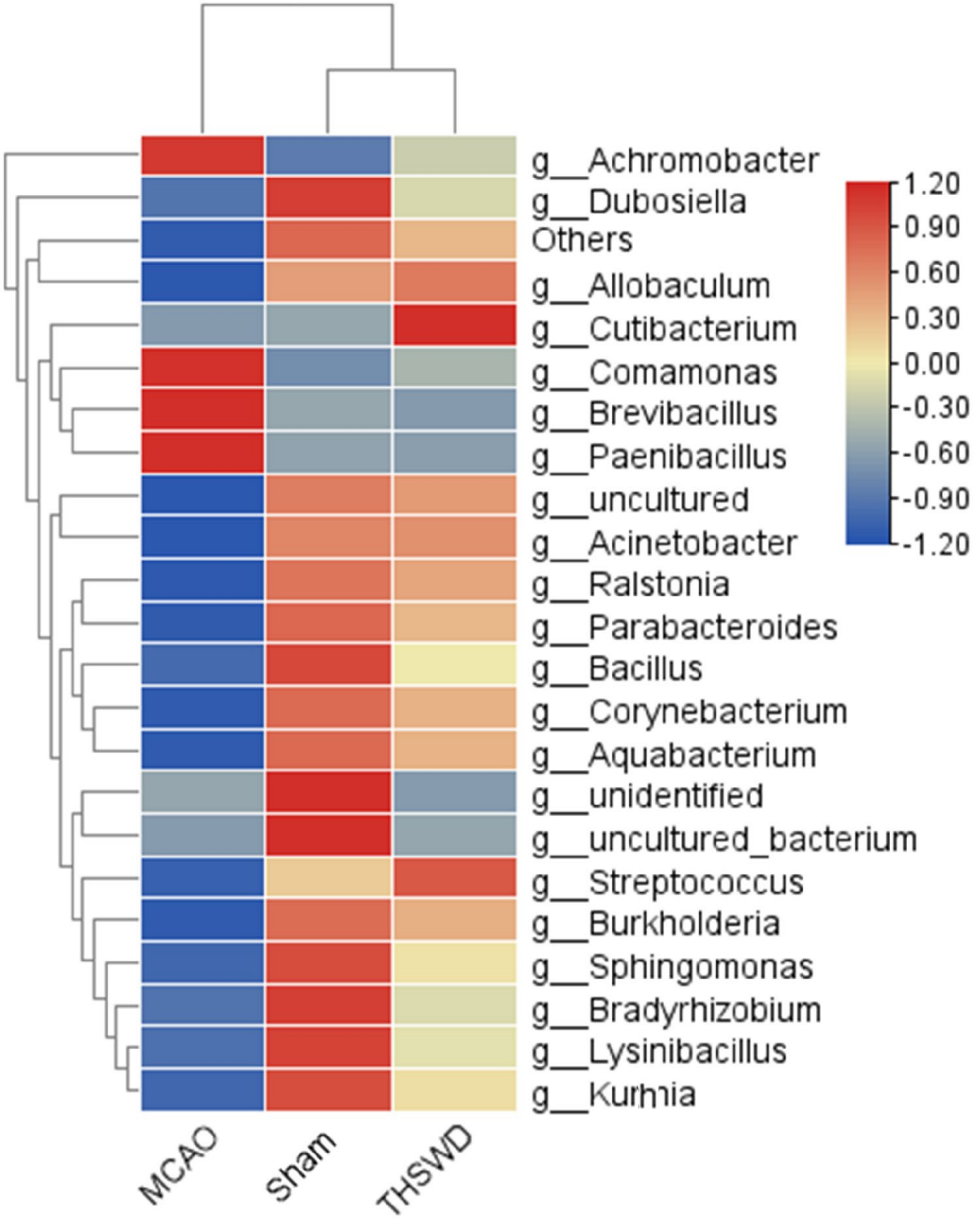
Effect of THSWD on relative abundance of gut microbiota at the level of genus in ischemic stroke mice

THSWD induced an increase in the alpha and beta diversity of our ischemic stroke mouse model. The abundance changes of intestinal flora in the three treatment groups were evaluated from the gate level. A total of 12 bacterial species were identified, among which *Firmicutes*, *Actinobacteria* and *Proteobacteria* were dominant. The abundance of *Firmicutes and Actinobacteria* were decreased while that of *Proteobacteria* was increased in the MCAO model. Following intervention with THSWD, the abundance of *Firmicutes and Actinobacteria* were increased while *Proteobacteria* contents was decreased (Fig. 4). At the level of genus classification, compared with the sham group, the relative abundance of *uncultured*, *Parabacteroides*, *Acinetobacter*, *Ralstonia*, *Allobaculum*, *Dubosiella*, *Corynebacterium*, and *Aquabacterium* in the model group was lower while that of *Achromobacter*, *Comamonas*, *Brevibacillus* and *Paenibacillus* was higher. THSWD treatment had the opposite effect on the above bacterial contents (Fig. 5).

### Effect of THSWD on intestinal barrier destruction in mice with ischemic stroke

**Fig. 6:**
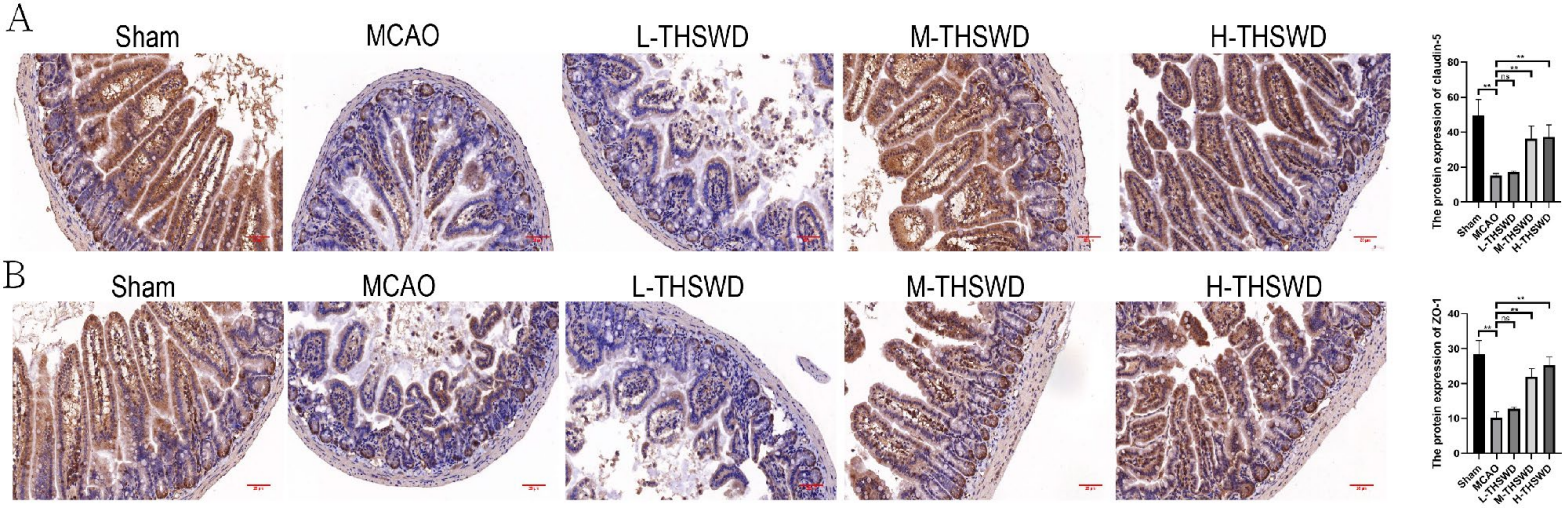
Effect of THSWD on intestinal barrier destruction in ischemic stroke mice. (**A**) Immunohistochemical detection of claudin-5 expression in intestine. (**B**) Immunohistochemical detection of ZO-1 expression in intestine. Magnification: X400. Scare bar = 20 μm. ^**^P < 0.01

**Fig. 7:**
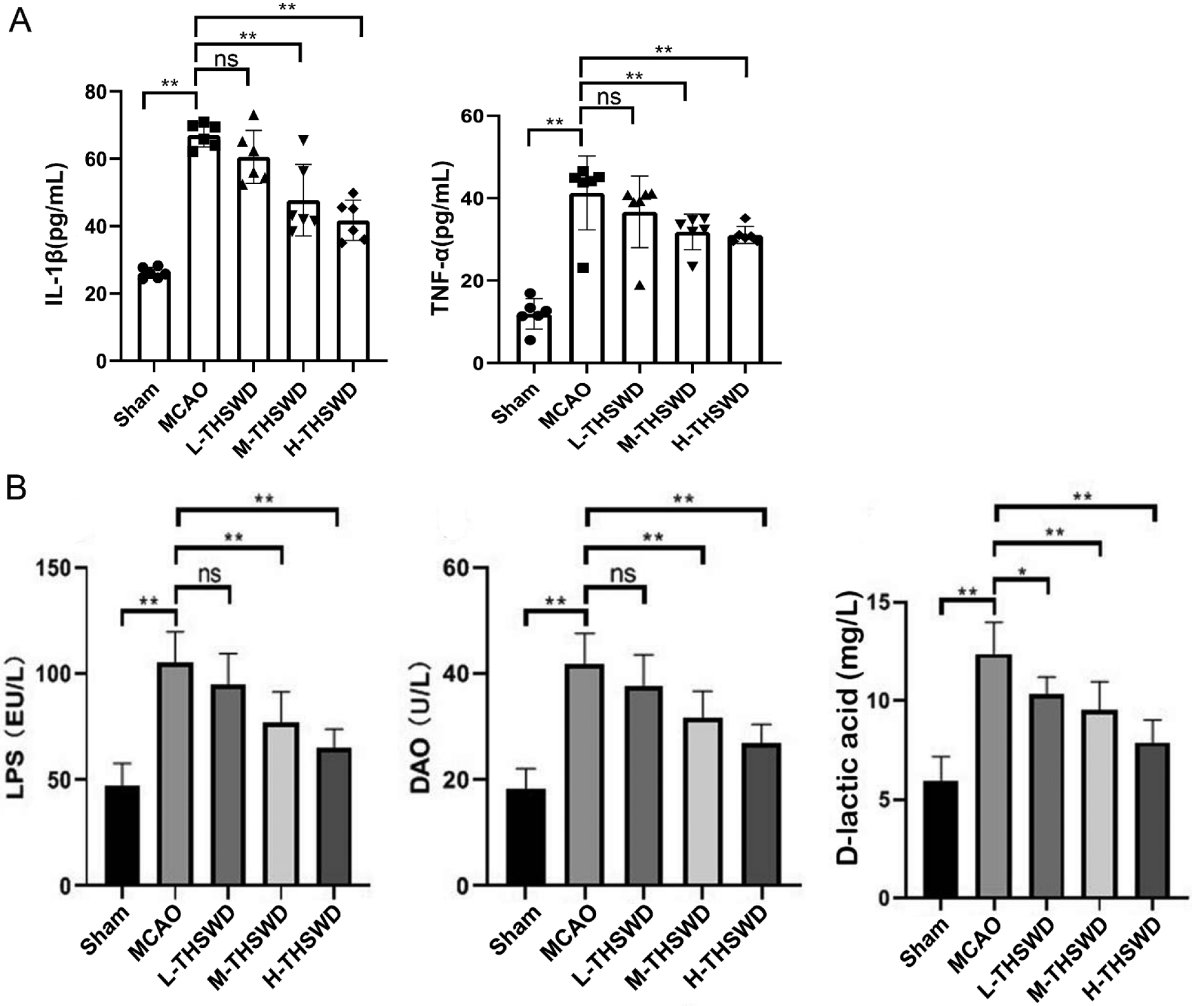
Effect of THSWD on expression of inflammatory cytokines in ischemic stroke mice(mean ± SD, *n* = 6). (**A**) The expression of IL-1β and TNF-α in brain tissue of ischemic stroke mice. (**B**) The expression of LPS、, DAO and D-lactic acid in serum of ischemic stroke mice. ^*^P < 0.05, ^**^P < 0.01

The barrier function of intestinal mucosa is also critical in maintaining the balance of intestinal flora. We initially examined the expression of tight junction proteins in colon samples of mice from each group. The levels of Claudin-5 and ZO-1 in the colon of mice in the MCAO group were lower than in the sham group (*P* < 0.01), and the expression levels of Claudin-5 and ZO-1 in the colon tissues of mice in the high- and medium-dose THSWD group were increased compared with the model group (*P* < 0.01)(Fig. 6). In addition, following the damage of the intestinal barrier, intestinal flora and their metabolites can migrate from the intestinal tract to the blood circulation (designated intestinal flora translocation), resulting in a series of complications, such as endotoxemia and infection. Therefore, DAO, LPS and D-lactate were analyzed as intestinal flora translocation indexes. Compared with the sham group, the contents of serum DAO, LPS and D-lactate in the MCAO group were significantly higher (*P* < 0.01), supporting the theory that ischemic stroke destroys the intestinal barrier and promotes intestinal microbial translocation. After treatment with THSWD, serum DAO, LPS and D-lactic acid levels were significantly decreased (*P* < 0.01, *P* < 0.05)(Fig. 7B). Our results clearly suggest that THSWD effectively protects the intestinal barrier, thus weakening bacterial translocation.

### Effect of THSWD on inflammatory cytokine expression in mice with ischemic stroke

ELISA detection of inflammatory factors in brain tissue revealed significantly higher IL-1β and TNF-α contents in the MCAO group relative to the sham group (*P* < 0.01). However, the expression of inflammatory factors was significantly reversed in high and middle dose groups of THSWD(*P* < 0.01;Fig. 7A).

### Effect of THSWD on microglial cell polarization in mice with ischemic stroke

**Fig. 8:**
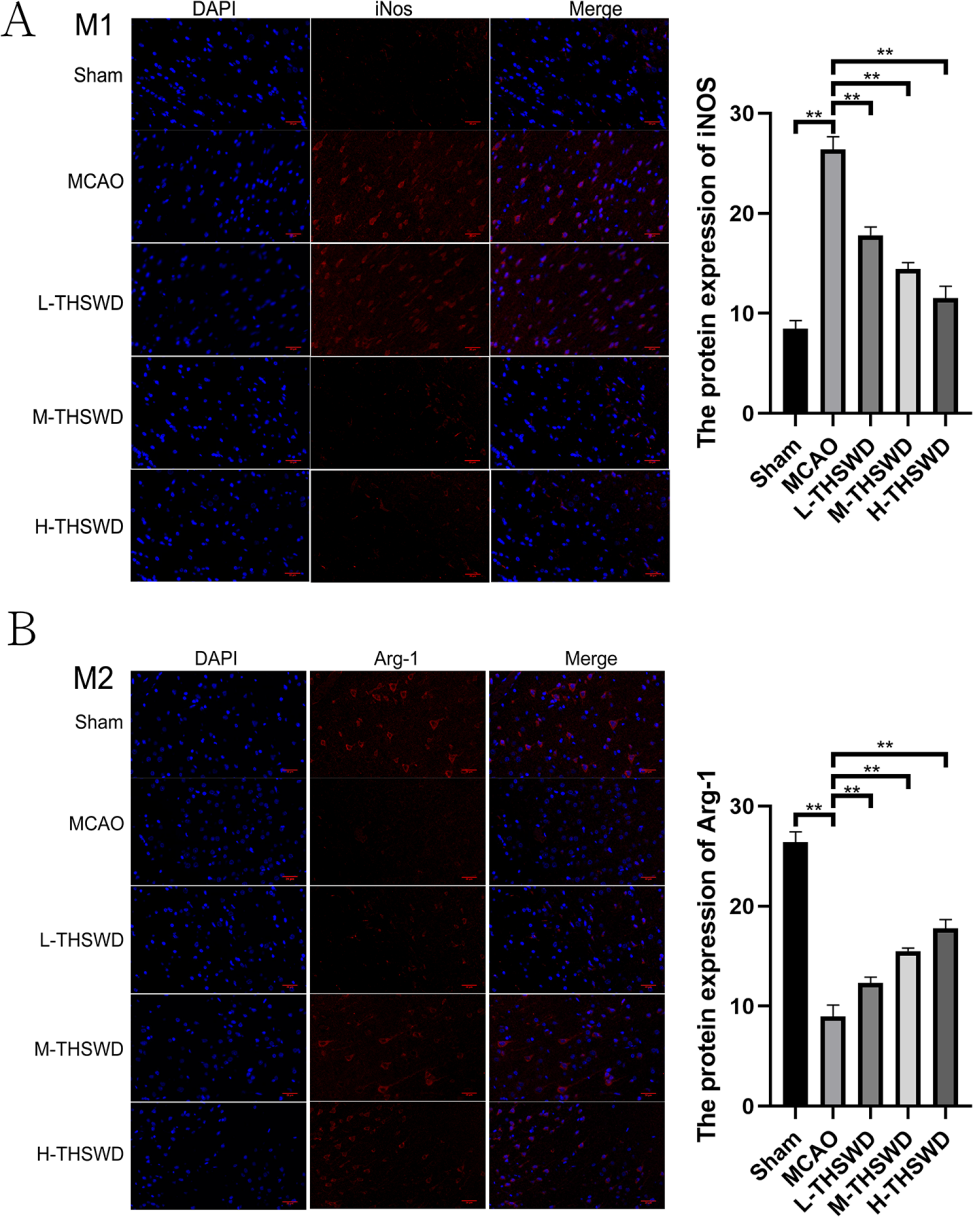
Effect of THSWD on microglial cell polarization in ischemic stroke mice (**A**) iNOS expression in mice brain. (**B**) Arg-1 expression in mice brain. Magnification: X400. Scare bar = 20 μm. ^**^P < 0.01

In the results of immunofluorescence staining, the sham group served as the reference control, the number of iNOS-positive cells of M1 microglia, an activation marker of the pro-inflammatory phenotype, was increased in the MCAO group (*P* < 0.01). The number of iNOS-positive cells in each treatment group was significantly lower than that of the MCAO group (*P* < 0.01)(Fig. 8A).

Relative to the sham group, the number of Arg-1-positive cells of M2 microglia, a marker of the anti-inflammatory phenotype, was decreased in the MCAO group(*P* < 0.01). Relative to the MCAO group, the number of Arg-1-positive cells in each THSWD group was increased (*P* < 0.01)(Fig. 8B).

### Effects of THSWD on TLR-4 and NF-κB levels in mice with MCAO-induced ischemic stroke

**Fig. 9:**
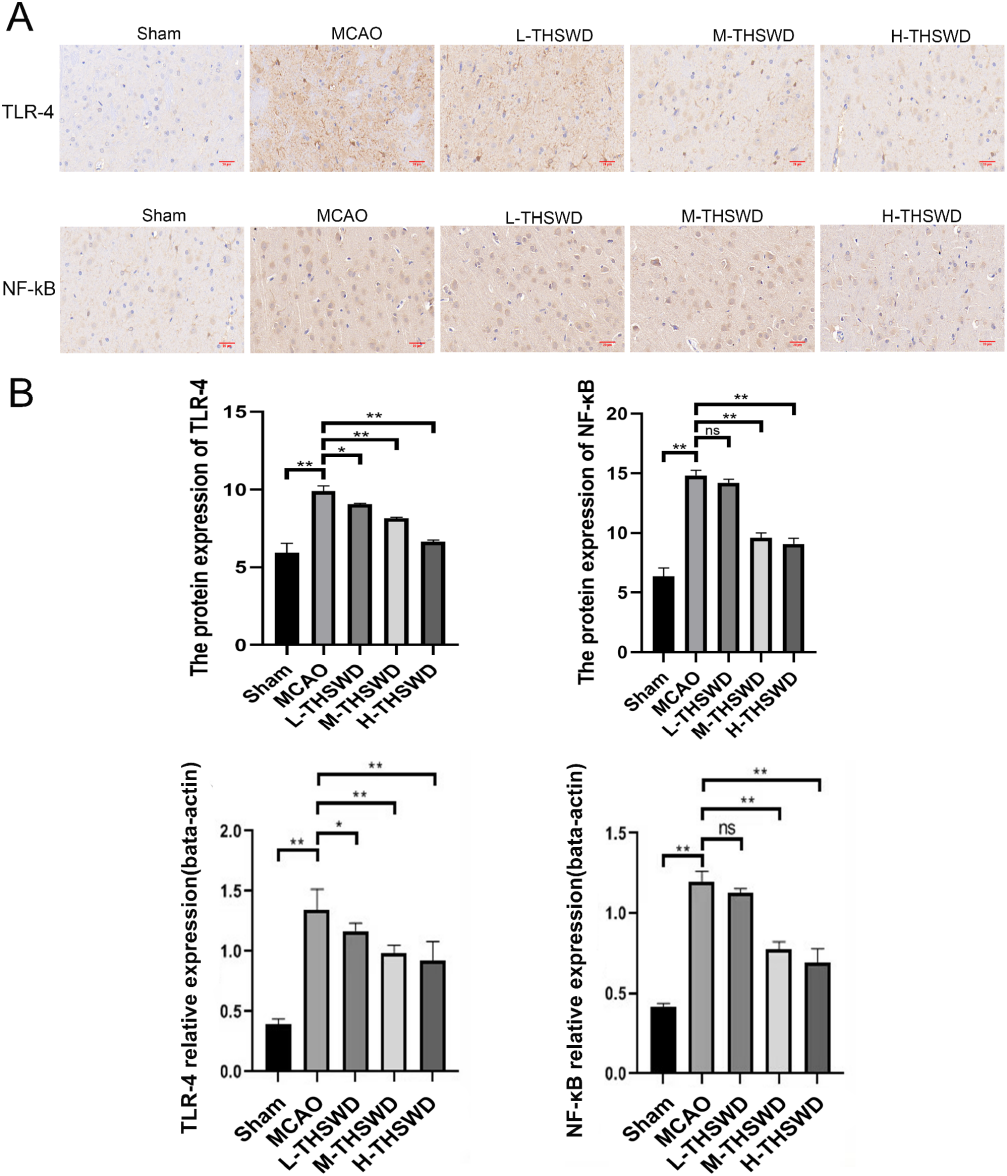
(**A**) Immunohistochemical analysis of TLR-4、, NF-κB protein expression in the brain tissue. (**B**) qRT-PCR analysis of TLR-4、, NF-κB mRNA expression in the brain tissue (mean ± SD, *n* = 6). Magnification: X400. Scare bar = 20 μm. ^*^P < 0.05, ^**^P < 0.01

Immunohistochemistry experiments disclosed higher TLR-4 and NF-κB protein expression in the MCAO group compared to the sham group (*P* < 0.01). TLR-4 protein levels in all three THSWD treatment groups were lower than that in MCAO group (*P* < 0.01, *P* < 0.05). Moreover, NF-κB protein levels in the middle- and high- dose THSWD treatment groups were decreased (*P* < 0.01) (Fig. 9A).

In qRT-PCR analysis, TLR-4 and NF-κB mRNA expression levels in the MCAO group were significantly higher than those in sham operation group (*P* < 0.01) and THSWD decreased the expression of these proteins in MCAO(*P* < 0.01, *P* < 0.05)(Fig. 9B).

## Discussion and conclusion

Diversity is one of the main parameters reflecting the stability, balance and resilience of intestinal flora. Diversification of intestinal microflora is beneficial to health. Ischemic stroke induces a series of changes in the diversity and structural characteristics of intestinal flora, along with impairment of function [26]. Moreover, fecal bacteria and organic acid concentrations are significantly altered in patients in association with metabolic and inflammatory markers of ischemic stroke [27]. Recent studies have reported a decrease in diversity of intestinal flora, significant reduction in abundance of specific bacteria (such as *hard-walled bacteria*, *Lactobacillus* and *Clostridium*), and increase in *Escherichia* *coli*, *Bacteroides*, *Megamonas*, *parasitobacteria* and *rumen* *cocci * in patients with ischemic stroke [28, 29]. The changes of intestinal flora composition were further confirmed in our MCAO model rats [30]. In these years, accumulating studies have demonstrated that damage of intestinal flora is a risk factor of prognosis after stroke. Bacteria in the intestinal tract regulate neuronal function by producing neuroactive compounds, such as neurotransmitters and hormones. Behavior after ischemic stroke is significantly altered [31]. In addition, intestinal flora and their products affect host metabolism and immune status as well as the neural network in ischemic brain [32]. Herefore, maintenance of normal intestinal physiological functions may play a positive role in the treatment and prognosis of ischemic stroke.

We explored the effect of THSWD on ischemic stroke on intestinal flora imbalance in mice. 16s rDNA sequencing was employed to evaluate changes in fecal microbiota of each group. Our results showed that the alpha and beta diversity of intestinal flora in the model group was decreased and abundance of *Firmicutes* and *Actinobacteria*, *uncultured*, * Parabacteroides*, *Acinetobacter*, *Ralstonia*, *Allobaculum*, *Dubosiella*, *Corynebacterium*, *Aquabacterium* was decreased while that of *Proteobacteria* and *Achromobacter*, *Comamonas*, *Brevibacillus* and *Paenibacillus* was increased, clearly indicating that ischemic stroke triggers an imbalance in the intestinal microbiota in mice, consistent with previous findings [33]. THSWD induced an increase in alpha diversity and beta diversity of intestinal flora under ischemic conditions. *Parabacteroides distasonis*, a gram-negative anaerobe and member of the core microbiome of the human intestinal tract, is located on the surface of human duodenal epithelium. The host benefits from the presence of *parasitobacteria* and *Parabacteroides* plays a role in regulation of immunity. Earlier studies have shown that *P.distasonis* stimulates expression of IL-10 in human CD4 + CD25 + T cells and mouse IL-10 + FoxP3 + Tregs to regulate the immune response [34]. Simultaneously, *Parabacteroides* is significantly associated with anti-inflammatory factors and exerts a protective effect on intestinal barrier permeability through anti-inflammatory activity [35]. Moreover, *P.disasonis* promotes the production of succinic acid and secondary bile acid in the mouse intestine, thus activating intestinal gluconeogenesis and protecting intestinal permeability [36]. *Allobaculum* is a gram-positive bacterium with cells that are rod-shaped and arranged in pairs or chains. Related studies have shown that *Allobaculum* is a potential probiotic that can utilize probiotics and confer benefits to the host, and also acts as an active lactic acid user and butyrate producer in mice [37, 38]. Butyric acid specifically inhibits regulatory T cell histone deacetylase through suppressing G protein-coupled receptors to achieve anti-inflammatory effects. *Dubosiella*, a member of Phaeophyta, is reported to be highly correlated with mRNA expression of Nrf-2, HO-1 and IL-10 in colon tissue, showing that a potential role in enhancing antioxidant and anti-inflammatory ability [39]. SCFAs are essential for integrity of the intestinal barrier. Previous studies suggest that increasing SCFA levels can reduce colitis through suppression of pro-inflammatory cytokines [40]. *Parabacteroides*, *Allobaculum * and *Dubosiella* have been shown to produce SCFAs. Notably, SCFA-producing bacteria can effectively protect the mucous membrane from pathogen damage by providing colon cell nutrition and reducing inflammation, protect the intestinal barrier and maintain intestinal immune homeostasis, which are beneficial for the host [41, 42]. Based on the above findings, we speculated that THSWD could regulate intestinal microflora homeostasis in mice with ischemic stroke by increasing diversity and improving species composition of the intestinal microbiome, ultimately reversing ischemia-induced dysbiosis to a certain extent.

Transmembrane barrier proteins (such as claudin-5) and cytoplasmic scaffold proteins (such as ZO-1) are markers of intestinal mucosal barrier integrity. In this study, levels of claudin-5 and ZO-1 in colon tissue of the MCAO group were significantly lower than those of the sham group, consistent with the report of Chen et al. [43]. The THSWD effectively prevented intestinal permeability mediated by ischemic stroke and reversed the expression patterns of claudin-5 and ZO-1, confirming the significant therapeutic efficacy of this traditional Chinese medicine.

Under conditions of increased intestinal permeability and barrier damage, intestinal flora and their metabolites migrate from the intestinal tract to the systemic circulation, resulting in a series of disease complications, such as endotoxemia and infection, a phenomenon known as intestinal flora translocation [44]. The intestinal products LPS, D-lactic acid and DAO enter the blood circulation through the damaged intestinal barrier and can therefore serve as indexes reflecting intestinal permeability and bacterial translocation [45]. Therefore, we further studied the translocation of intestinal flora by detecting the levels of DAO, endotoxin and D-lactic acid. The contents of LPS, D-lactic acid and DAO increased significantly after cerebral ischemia, suggesting that ischemic stroke destroyed the intestinal barrier and led to intestinal microbial translocation. After treatment with THSWD, the contents of LPS, D-lactic acid and DAO decreased significantly, indicating that THSWD can effectively weaken bacterial translocation and endotoxin in systemic circulation. Therefore, the above results show that THSWD can improve the increase of intestinal permeability, the destruction of intestinal barrier and bacterial translocation caused by cerebral ischemia.

When LPS accumulates in the intestinal mucosa, it will activate macrophages and lead to neutrophil overflow. At the same time, its mediated inflammatory response will also lead to the destruction of the intestinal barrier, which in turn causes excessive pathogenic bacteria and LPS to circulate into the bloodstream, triggering an inflammatory cascade reaction [46]. Similar to the intestinal barrier, a blood-brain barrier structure exists in brain tissue. LPS can enter the brain tissue along with the blood circulation through the damaged blood-brain barrier, causing further brain damage [47]. LPS entry into the brain occurs via two pathways: (1) passing through the weak area of the blood-brain barrier and spreading to brain tissue, and (2) binding to serum proteins in the blood-brain barrier, followed by absorption by endothelial cells with specific transport capacity [48]. The blood-brain barrier (BBB) is a selective permeable barrier between the blood and central nervous system, which effectively regulates the inflow and outflow of substances, protects nerve cell function and maintains brain homeostasis. The blood-brain barrier is broken and harmful substances such as LPS flow into the brain, affecting inflammation and immune response [49]. In this study, we further showed a protective effect of THSWD against disruption of the blood-brain barrier after ischemic stroke.

Microglia play a vital role in maintaining and supporting the normal physiological and metabolic state of neurons in multiple ways and participate in neuronal regulation and protection, thus maintaining the dynamic stability of the central nervous system [50]. In ischemic stroke, microglia are activated and then recruited to the brain lesion area, where the number of microglia increases [51]. At the same time, the cells become smaller, with shorter branching and phagocytosis, altering shape from “branching” to “shrub-like” and subsequently to “amoeba-like”. Activated microglia are classified into M1 and M2 subtypes [52]. M1 microglia have a pro-inflammatory effect and are induced by IFN-γ and LPS. The main markers are CD11b and iNOS, which aggravate brain injury and promote nerve cell death and blood-brain barrier destruction by releasing a large number of pro-inflammatory factors, such as interleukin-IL-1β, tumor necrosis factor-α (TNF-α) and IL-23 [53]. Recent studies have shown that salvianolic acid C (the main component of Salvia miltiorrhiza) inhibits polarization of microglia, promotes endothelial tubule formation and performs nerve repair function of cerebral ischemia [54]. The main markers of M2 microglia are CD206 and Arg-1, which inhibit inflammation by secreting IL-10, and play a role in reducing injury while promoting tissue repair and neuroprotection. The TLR4/NF-κB signal pathway is closely related to ischemic stroke. As a harmful signal stimulus, LPS binds to TLR4, an innate immune recognition molecule on central microglia, resulting in transmission of transmembrane information [55]. NF- κB is the main participant in both non-specific and specific immune responses at the core of TLR4 signal transduction. Recent studies have shown that Schisandra B inhibits the harmful consequences of Imax R injury and achieves a neuroprotective effect by inhibiting TLR4/NF- κB signaling [52]. Blockage of downstream transduction of the TLR/NF- κB pathway and regulation of the proportion of activated M1/M2 microglia and cytokine expression after cerebral ischemia has been shown to minimize tissue injury and protect brain [56]. Therefore, block the activation of NF- κB signal pathway, control of microglial activation, reduction of inflammatory mediators and inhibition of inflammatory response are critical in reducing cerebral ischemic injury and promoting the recovery of neurological function. Data from the current study revealed that the traditional Chinese prescription, THSWD, reduces activation of microglia induced by ischemic stroke, inhibits the release of pro-inflammatory factors (IL-1β and TNF-α) in brain tissue, and suppresses the expression of TLR-4 and NF- κB at both protein and gene levels.

In summary, THSWD may have a protective effect on inflammatory injury after ischemic stroke by regulating the richness and diversity of intestinal flora, protecting intestinal barrier, reducing the release of endotoxin, inhibiting TLR4/NF-κB pathway, regulating the activation and phenotype of microglia, and affecting the release of inflammatory factors.

## Data Availability

The original contributions presented in the study are included in the article/Supplementary Material. Further inquiries can be directed to the corresponding authors.

## Abbreviations

THSWD: Tao Hong Si Wu Decoction
SPF: Specefic pathogen Free
ZO-1: occludens-1
LPS: lipopolysaccharide
DAO: diamine oxidase
TNF-α: tumor necrosis factor-α
IL-1β: interleukin − 1β
TLR-4: toll-like receptor-4
NF-κB: nuclear factor kappa B
MCAO: middle cerebral artery occlusion
